# Does gender matter? The impact of gender and gender match on the relation between destructive leadership and follower outcomes

**DOI:** 10.1186/s40359-025-02566-7

**Published:** 2025-03-18

**Authors:** Mats Reinhold, Andreas Stenling, Britt-Inger Keisu, Robert Lundmark, Susanne Tafvelin

**Affiliations:** 1 Department of Psychology, Umeå university, 901 87 Umeå, Sweden; 2 Department of Sociology, Umeå university, Umeå, Sweden; 3 Umeå Center for Gender Studies, Umeå University, Umeå, Sweden; 4 Department of Health, Education and Technology, Luleå University of Technology, Luleå, Sweden; 5 Department of Sport Science and Physical Education, University of Agder, Kristiansand, Norway

**Keywords:** Destructive leadership, Gender match, Follower, Work performance, Well-being

## Abstract

**Background:**

Destructive leadership has been linked to negative consequences for both organizations and followers. Research has also shown that leader gender affects follower perceptions of leadership behavior and follower outcomes [1–3]. However, knowledge is limited as to whether this also applies to destructive leadership [4]. This study aims to combine gendered organization theory with destructive leadership research to investigate the role that gender plays in the relation between destructive leadership behavior and follower outcomes.

**Methods:**

The data were collected in collaboration with Statistic Sweden. It is a representative sample from the working population in Sweden. We used a two-wave survey design and included 1,121 participants in the analysis.

**Results:**

The results from structural equation models indicated that destructive leadership has negative consequences for follower burnout, job satisfaction, and turnover intention 6 months later. The results also showed that followers reported a greater intention to leave the organization if the leader was the same gender and used destructive leadership.

**Conclusions:**

Our study contributes to destructive leadership research by showing that the gender of both the leader and follower matters for the relation between destructive leadership behavior and follower outcomes. Additionally, our study makes a theoretical contribution by integrating a gender research perspective into destructive leadership research.

## Background

The consequences of destructive leadership have been well documented in the literature [5, 6]. Research has shown negative effects of destructive leadership, including reduced productivity, counterproductive work behavior, decreased well-being, absenteeism, and increased turnover intention among employees [5, 6]. Destructive leadership is a multidimensional construct that encompasses the behaviors of leaders that affect and harm followers and organizations [7]. Studies have shown that destructive leadership is a common phenomenon, with prevalence estimates ranging from 36 to 61% [8, 9], and there is an increasing amount of research on this topic.

While research on constructive leadership has demonstrated that gender significantly affects how various leadership styles are perceived and how they impact followers [2, 10–15], studies examining the role of gender in destructive leadership remain limited. This is surprising given the extensive body of research on gender and leadership, some of which spans over 50 years [2]. Since women began entering the labor market and assuming leadership positions, scholars have been keen to understand how and why gender influences the behaviors of male and female leaders, as well as how these behaviors are evaluated by others [2, 10, 16]. Feminist scholars have revealed the gendered nature of leadership, the strong association between masculinity, men, and leadership, and how societal perceptions often view women and femininity as deficient in relation to these constructs [17, 18]. They argue that leadership consists of a complex interplay between gender and power [16, 17]. An interplay that may also influence gendered perceptions of who enacts a destructive leadership [19, 20].

This idea is further supported by Stempel and Rigotti’s pioneering study [4], which showed that leader gender plays a role in employees’ perception of destructive leadership. According to their study, higher levels of destructive leadership at baseline were associated with higher levels of follower emotional exhaustion and somatic stress when the leader was male [4], suggesting that a gender perspective on destructive leadership is relevant and needs further investigation. The theoretical framework of the present study posits that leadership is a reciprocal interaction between leaders and followers [19, 20, 21]. Therefore, follower gender must be considered when examining the outcomes of destructive leadership. However, research is limited on the gender-match perspective between the leader and follower for the destructive leadership process [22, 23].

The purpose of the present study is to examine destructive leadership from a gender perspective. We do this by evaluating whether followers experience destructive leadership differently depending on the leader’s gender. Additionally, we examine whether leader gender and gender match (between the leader and follower) moderate the relation between perceived destructive leadership and follower job satisfaction, burnout, work performance, and turnover intention. The main contributions of this study lie in clarifying the role of gender in these relations. First, in answer to the call from Kark and Buengeler [16], to incorporate theoretical perspectives, we build our hypotheses on gendered organization theory [21]. Based on social constructivist principles, gendered organization theory posits that organizations are not gender neutral, and it examines how gender dynamics are embedded in organizational structures, workplace culture, and interactions between leader and follower. Furthermore, it suggests that leader and follower identity can affect followers’ experience of gender and gendered behaviors, such as destructive leadership. Several scholars within this field have clarified how gendered processes produce inequalities [21, 25]. Thus, we expand the understanding of destructive leadership and gender in the lens of more recent feminist approaches in leadership research [24].

Second, we extend previous research on destructive leadership and gender by highlighting the role of follower gender. Leader gender affects how followers perceive their destructive behaviors [3]. In dynamic relationships, the gender of followers should also be considered. Hence, we introduce a gender-match perspective to understand how leader and follower gender combined affect the consequences of destructive leadership.

## Destructive leadership and follower outcomes

Schyns and Schilling [5] defined destructive leadership as a “process in which, over a longer period of time the activities, experiences and/or relationships of an individual or the member of a group are repeatedly influenced by their supervisor in a way that is perceived as hostile and/or obstructive” (p. 14) The perception of a behavior as destructive is key and in line with a number of previous studies; we view the concept of destructive leadership as a multidimensional phenomenon that includes both active behaviors, such as threats, unfairness, punishment, and overdemands, and passive behaviors, such as absence, avoiding making decisions, and being messy or unclear [26, 9, 27, 28, 29].

The conservation of resource [COR] theory [30, 31] provides a theoretical framework for exploring how destructive leadership relates to follower outcomes. According to COR theory, people strive to gain, maintain, or protect valued resources to ensure self-preservation [32]. People’s reactions to preserving personal resources determine their reactions in stressful situations [33]. Hobfoll [29] defined these resources as objects (e.g., house or car), conditions (e.g., job security or social support), energies (e.g., attention or time), and personal characteristics (e.g., self-efficacy or self-esteem). When resources are threatened or lost, people will experience a depletion of energy that leads to work fatigue and stress. The loss of resources can ultimately affect follower outcomes, such as well-being and performance. Destructive leadership from a COR perspective can be seen as an interpersonal stressor directly reducing well-being and as a loss of social resources regarding access to leader support [34].

Thus, to measure the level of follower outcomes due to destructive leadership behavior, we use several relevant indicators. In the present study, we use job satisfaction and burnout as indicators of followers’ well-being. Job satisfaction is defined as “the pleasurable emotional state resulting from the appraisal of one’s job as achieving or facilitating the achievement of one’s job value” [35]. Burnout is defined as a feeling of emotional exhaustion, which affects social interactions and causes negative feelings and attitudes [36]. Work performance is used as an indicator and is defined by Motowildo et al. [37] as “the aggregated value to the organization of the discrete behavioral episodes that an individual performs over a standard interval”. We used turnover intention as an indicator that refers to followers’ intention to leave a job. Previous research has shown negative consequences for followers’ well-being [5, 38,–42], work performance [5, 7, 29, 43, 44], and turnover intention [45–48]. Based on our theoretical suggestions and empirical findings, we propose the following four hypotheses:

### Hypothesis 1

1a: Destructive leadership will have a positive relation with followers’ level of burnout 6 months later.

### Hypothesis

1b: Destructive leadership will have a negative relation with followers’ level of job satisfaction 6 months later.

### Hypothesis

1c: Destructive leadership will have a negative relation with followers’ work performance 6 months later.

### Hypothesis

1d: Destructive leadership will have a positive relation with followers’ turnover intentions 6 months later.

## Destructive leadership and gender

Leadership can be understood as a social process between leader and followers. From a gendered organization theory perspective, leadership behaviors are based on gendered assumptions. This approach challenges the conventional attribution of specific traits and behaviors to men and women, arguing instead that assumptions about behaviors, characteristics, and attributes are culturally ascribed to femininity and/or masculinity [16, 17, 49, 50]. Idealized representations of traits and tendencies, for example leadership behaviors, that are linked to a certain sex may not reflect the reality, they function within the discourse to significantly influence how we perform, and are anticipated to perform, our gender identities [17, 20]. In general, these assumptions often associate women with feminized or communal behaviors [e.g., understanding, good-natured, caring, and warmth; 18], whereas men tend to be associated with masculinized or agentic behaviors [e.g., competitiveness, independence, confidence, and competence; 18]. These types of unexamined beliefs and images that is associated to different gender both produce and perpetuate stereotypical associations about leaders and leadership in interactions between the leader and follower [21, 51]. Furthermore, idealized images of leadership behaviors put significant pressure on both men and women, even when these pressures are subtle [17]. However, male leaders appear to have greater flexibility in adopting behaviors traditionally associated with femininity, while female leaders tend to face more constraints in engaging in behaviors typically associated with masculinity [52]. Female leaders have for example less ability to behave badly and still be perceived as credible leaders compared to male ones [20] and some scholars [17] suggest that the domination by men in leadership positions have been going on for so long that it is a conflate between doing masculinity, doing work, and doing leadership. In order to be perceived as competent both men and women need to adopt to these gendered expectations. Following this line of argument, based on gendered organization theory [21], we suggest that the strong association between male leaders, agentic, and destructive leadership behaviors (e.g., threatening followers or avoiding interactions with employees), combined with the narrower scope for female leaders to engage in destructive leadership while still remaining credible as a leader, results in male leaders being perceived as more destructive than female ones.

Although interactions often serve to reinforce existing inequalities, whether deliberate or not, interactions between leaders and followers can foster equality by actively striving for gender awareness, inclusion, and diversity [21]. Empirical findings from the few existing studies are mixed. Some studies have shown that male leaders are perceived as more destructive than female ones [1, 2, 53], whereas other results show no difference [4]. However, the combined arguments based on the association between masculine-marked behaviors, destructive leadership, and stereotypic images of a credible leader, together with previous empirical findings, suggest the following hypothesis:

### Hypothesis 2

Followers perceive male leaders as more destructive than female leaders.

Structures of dominance and submission in an organization are based not only on leader–follower relations but also on gender hierarchies between men and women in our society. Organizations are often seen as gender-neutral bureaucratic entities [51]. However, there is a reciprocal relation between societal and organization cultures. Organization cultures have a great variety and are contextual, and we define the concept as the sum of attitudes, behaviors, images, values, and beliefs within an organization [21]. Hence, activities such as decision-making, along with implicit and explicit workplace behaviour, rules, and organizational hierarchies, are shaped and challenged by power dynamics within the culture of an organization. Hierarchical power and status in organizations are typically linked to male leaders [54, 55], who are perceived as superior [10, 13, 51]. This combination of formal leadership power and informal power in a patriarchal society gives male leaders an advantage over female leaders [20, 21, 51]. Therefore, we assume that when a male leader engages in destructive leadership, such as punishing or ridiculing a follower, not attending meetings, and avoiding followers, his behavior has greater influence on follower outcomes. This is because his destructive behavior is associated with more power and agency, according to both organization culture and societal norms, compared to female destructive leaders. Based on gendered organization theory [21], regarding the intertwined relation between hierarchies in society, organization culture, and gender-marked behaviors, we argue that male leaders’ destructive leadership affects follower outcomes more than that of female leaders.

To the best of our knowledge, only a few studies have investigated whether leader gender affects the relation between destructive leadership and follower outcomes [e.g., 56, 53, 2]. Stempel and Rigotti [4] found that, compared with female leaders, followers rated the effect of destructive leadership on their somatic stress and emotional exhaustion more strongly if the leader was male. We want to know if this holds true for follower burnout, job satisfaction, work performance, and turnover intention as well. Based on the above implications, we propose the following hypothesis for destructive leadership and follower outcomes:

### Hypothesis 3

Leader gender moderates the negative relations between destructive leadership and follower burnout, job satisfaction, work performance, and turnover intention 6 months later, such that the relations are stronger for followers with a male leader compared to followers with a female leader.

The leader and follower both influence leadership behaviors [20]. Leadership comprises different behaviors and is an ongoing process that takes place in relation to others and varies depending on contexts. Sociocultural norms and expectations shape the image of the ideal leader and leadership [20, 21, 49]. Additionally, gendered norms influence how both leaders and followers perceive each other in a leadership process [21, 57]. The Swedish labor market is highly gender segregated, even to a higher extent compared to the rest of the European Union [EU; 58]. According to gendered organization theory, a gender-segregated labor market influences how followers perceive a leader [51, 59]. For example, in sectors dominated by women, most leaders are also women, and it is conceivable that long-term gender segregation, where most followers have women in leadership positions, could affect follower perceptions of leadership norms [20, 21, 60]. In gender-segregated contexts where female leaders are in the majority, they are perceived to be the norm and the familiar. Hence, followers are used to female leaders, and their agency and power are not compared to male leaders as they are in male-dominated contexts and sectors [59]. According to a gender-match perspective, people tend to identify more easily with others with similar characteristics, background, and cultures, suggesting a stronger relationship between same-gender leader–follower dyads compared to different-gender dyads [61, 62]. For example, in gender-segregated sectors, same-gender dyads between the leader and follower would be expected. A gender-match perspective suggests that the female follower has a stronger identification with her female leader and looks up to her leader, and her feelings of betrayal when she becomes a victim of destructive leadership behavior make her more vulnerable compared to a weaker relationship between different gender dyads. A similar identification effect occurs if the leader and follower are men.

Leadership research has shown that the gender of both leaders and followers jointly influences follower perceptions of leaders [11, 22, 62, 63]. Powell and Butterfield [61] and Stoker et al. [62] showed that leaders of the opposite gender are preferred less and rated as less effective than leaders of the same gender, regardless of the gender composition of the dyad. To the best of our knowledge, there is no existing research on the interplay between destructive leadership and gender match. We propose that destructive leadership has a stronger effect in a same-gender leader–follower composition than in a mixed-gender composition:

### Hypothesis 4

The gender composition of the leader–follower dyad will affect how followers are impacted by destructive leadership. More specifically, a matching gender composition between the leader–follower will have a stronger negative effect on follower outcomes compared to no gender match.

## Methods

### Sample and procedure

We used a randomly selected sample from both the private and public sectors, equivalent to the Swedish labor market demography (*N* = 3000). The sample was drawn from the Swedish Occupational Register, an annually updated record of all employees in Sweden. Statistics Sweden administered the questionnaires. The first questionnaire (T1) was distributed and returned by post. After three reminders, 1,121 participants returned the questionnaire, resulting in a response rate of 37.4%. A follow-up questionnaire (T2) was sent 6 months later via postal and e-mail (with a link to a web-based questionnaire to enable a higher response rate) to the respondents at T1. A total of 800 participants answered the questionnaire after three reminders.

A total of 744 females and 377 males were included (total *n* = 1121), with a mean age of 47.8 years for females (*SD* = 10.9) and 47.9 years for males (*SD* = 11.6). The highest completed education levels among the participants were 4.8% for primary school, 31.5% for secondary school, and 58% for university. Another type of education was reported by 5.6%. For employees with a female leader, 61.2% and 38.8% had a male leader. Participants had worked under their present immediate leader for 3.1 years on average (*SD* = 3.4), and 82.6% of the participants had interacted directly with their leader at least once per week or more.

### Measures

#### Destructive leadership

We assessed followers’ perceptions of their leader’s destructive leadership using Destrudo-L [64]. Then, we included four subscales (two active destructive and two passive destructive), with four items each, in the global assessment of destructive leadership. The subscales included the following: Arrogant/Unfair, with an example item of “My boss treats people differently”; Threats/Punishments/Overdemands, with an example item of “My boss uses threats to get his/her way”; Passive/Cowardly, with an example item of “My boss does not show up among subordinates”; and Uncertain/Unclear/Messy, with an example item of “My boss shows insecurity in his/her role.” Responses were given on a 6-point Likert scale ranging from 1 (*never/almost never*) to 6 (*always*). The reliability (ω) of the destructive leadership scale [65], with the four subscales, was 0.95. The reliability estimates of the four subscales were as follows: arrogant/unfair 0.91, threats/punishments/overdemands 0.88, passive/cowardly 0.89, and uncertain/unclear/messy 0.91.

#### Burnout

We measured work-related burnout using a 5-item subscale from the Copenhagen Psychosocial Questionnaire II [COPSOQ II; 66]. An example item is “During the last four weeks, how often have you felt worn out?” Responses were given on a 5-point Likert scale ranging from 1 (*never/hardly ever*) to 5 (*always*). The reliability (ω) of the 5-item subscale in the present study was 0.90.

#### Job satisfaction

We used the COPSOQ II to assess followers’ job satisfaction and contains the item, “How pleased are you with your job as a whole, everything taken in?” Responses were given on a 5-point Likert scale ranging from 1 (*to a very low degree*) to 5 (*to a very high degree*).

#### Work performance

We used the 3-item individual task proficiency subscale from the Work Role Performance Scale [67] to measure followers’ work performance. An example item is “In what degree during the last month have you carried out the core parts of your job well?” Responses were given on a 5-point Likert scale ranging from 1 (*to a very low degree*) to 5 (*to a very high degree*). The reliability (ω) of the 3-item subscale in the present study was 0.85.

#### Turnover intentions

We measured followers’ turnover intentions using four items developed by Barham et al. [68]. An example item is “I am planning to look for a new job.” Responses are given on a 5-point Likert scale ranging from 1 (*strongly disagree*) to 5 (*strongly agree*). The reliability (ω) of the 4-item scale in the present study was 0.94.

### Statistical analysis

We estimated descriptive statistics and bivariate correlations using IBM SPSS Statistics, version 28.0.1.1(15). We used Mplus Version 8.4 [69] and the robust full information maximum likelihood estimator to estimate the structural equation models in our study. We used the robust maximum likelihood estimator to account for missing data and potential nonnormality [70, 71]. Similar to previous research [9], we specified the multidimensional Destrudo-L scale as a bifactor exploratory structural equation modelling, B-ESEM [71]. Specifying zero cross-loadings in traditional confirmatory factor analysis (CFA) models often render poor model fit and inflated factor correlations, which affect discriminant validity and lead to biased estimates in structural models. In exploratory structural equation modelling (ESEM) cross-loadings on nontarget factors are allowed. We specified them to be close to zero but not exactly zero, whereas target factor loadings were freely estimated. We used target rotation [73], which allows for the specification of target and nontarget factor loadings in a confirmatory manner. The bifactor model was specified with a global destructive leadership factor alongside four specific factors. Based on findings in studies indicating poor specificity of the ego-oriented/false subscale [9], no specific factor was included for this dimension. The ego-oriented or false items only contributed to the global destructive leadership factor. The outcome variables, such as burnout, job satisfaction, performance, and turnover intentions at T2, were specified as unidimensional constructs using CFA.

Following the initial examination of the measurement model, we estimated a full structural equation modeling (SEM) with the global and specific destructive leadership factors as predictors at T1, as well as burnout, job satisfaction, job performance, and turnover intentions as outcome variables at T2. In a second step, we examined the moderated effects of global destructive leadership at T1 on the follower outcomes at T2 as a function of (a) leader sex and (b) gender match between the follower and leader. We estimated latent variable interactions using the latent moderated structural equations [LMS method; 74]. We used the ESEM-within-CFA approach [72] to estimate latent variable interactions with a bifactor ESEM model. The bifactor ESEM model is re-expressed in a CFA framework using start values from the initial bifactor ESEM model. This re-expression was necessary because latent variable interactions cannot be combined with ESEM factors.

We examined the model fit of CFAs, ESEMs, and SEMs using multiple fit indices, such as the comparative fit index (CFI), the Tucker–Lewis index (TLI), the standardized root mean square residual (SRMR), and the root mean square error of approximation (RMSEA). CFI and TLI values of approximately 0.90 and SRMR and RMSEA values of approximately 0.08 indicated acceptable model fit [75]. The significance level was set to 0.05 in the structural models.

## Results

### Descriptive statistics

Table 1 displays the descriptive statistics, means, standard deviations, and correlations among the study variables. As in previous research destructive leadership [44] was shown to be positively correlated with follower burnout and negatively correlated with job satisfaction and turnover intention. However, in contrast to previous research [7], the correlation between destructive leadership and work performance was weak and not statistically significant.

**Table 1:** Descriptive statistics, latent variable correlations, mean and standard deviation

	M	SD	DLT1	WRPT2	BOT2	TOIT2	JST2
DLT1			1.000	ω=0.95			
WRPT2	4.16	0.62	-0.047	1.000	ω=0.85		
BOT2	2.71	0.88	0.294***	-0.277***	1.000	ω=0.90	
TOIT2	2.27	1.13	0.277***	-0.149***	0.481***	1.000	ω=0.94
JST2	3.85	0.87	-0.260***	0.310***	-0.492***	-0.548***	1.000

Note. BOT2 = burnout, TOIT2 = turnover intentions, WRPT2 = work performance, JST2 = job satisfaction, DLT1 = global destructive leadership, M = mean at T2, SD = standard deviation at T2

***p <.001, **p <.01, *p <.05

### Test of hypotheses

We first examined the longitudinal relations between destructive leadership and follower burnout, job satisfaction, work performance, and turnover intentions within a period of 6 months between T1 and T2. The model fit values for the measurement model are = 805.796, *df* = 369, *p <.*001, CFI = 0.977, TLI = 0.969, RMSEA = 0.032, 95% CI [0.029, 0.035], SRMR = 0.023, and the values for the structural model are = 1007.678, *df* = 453, *p <.*001, CFI = 0.972, TLI = 0.963, RMSEA = 0.033, 95% CI [0.030, 0.036], SRMR = 0.027. We found support for Hypothesis 1a, suggesting that destructive leadership is positively related to followers’ level of burnout (*β* = 0.294, *SE* = 0.035, *p <.*001) and support for Hypothesis 1b, proposing that destructive leadership is negatively related to followers’ level of job satisfaction (*β* = −0.260, *SE* = 0.038, *p <.*001). The relation between destructive leadership and followers’ work performance was weak and not statistically significant, contrary to Hypothesis 1c (*β* = −0.047, *SE* = 0.039, *p* =.229). We also found support for Hypothesis 1d, in that destructive leadership had a positive relation with followers’ level of turnover intentions (*β* = 0.277, *SE* = 0.035, *p <.*001; Table 2).

**Table 2:** Regression coefficients from the structural models predicting level at T2

	Standardized Estimate	Unstandardized Estimate	SE	*p*
Burnout				
DL → BOT2	0.294	0.248	0.035	0.000
*R* ^2^	14.7%			
Job satisfaction				
DL → JST2	-0.260	-0.228	0.038	0.000
*R* ^2^	8.9%			
Work performance				
DL → WRPT2	-0.047	-0.028	0.039	0.229
*R* ^2^	1.9%			
Turnover intentions				
DL → TOIT2	0.277	0.309	0.035	0.000
*R* ^2^	14.0%			

Model fit, χ^2^ = 1007.819, *df* = 453, *p* <.001, RMSEA = 0.033 (95% CI [0.030, 0.036]), CFI = 0.972, TLI = 0.963, SRMR = 0.027

BOT2 = burnout, TOIT2 = turnover intentions, WRPT2 = work performance, JST2 = job satisfaction, DL = global destructive leadership

Concerning gender, male leaders (*M* = 2.141, *SD* = 0.991, *p* =.532) were not perceived as more destructive than female leaders were (*M* = 2.101, *SD* = 1.026, *p* =.532; T). We used t-test to compare means for men and women. T-value was between − 0.06 and 1.316, and *p*-value was between 0.189 and 0.951.

Thus, Hypothesis 2 was not supported. Furthermore, leader gender did not moderate the relation between perceived destructive leadership and follower burnout, job satisfaction, work performance, or turnover intention (see Table 3). Hence, Hypothesis 3 was not supported.

**Table 3:** Regression coefficients from the structural models predicting level at T2, leaders’ gender as moderator

	Standardized Estimate	Unstandardized Estimate	SE	*p*
Burnout				
DL → BOT2	0.316	0.267	0.055	0.000
Leader gender → BOT2	0.027	0.046	0.038	0.478
INT → BOT2	-0.017	-0.029	0.035	0.632
*R* ^2^	14.6%			
Job satisfaction				
DL → JST2	-0.276	-0.243	0.046	0.000
Leader gender → JST2	-0.023	-0.041	0.038	0.548
INT → JST2	0.012	0.022	0.042	0.769
*R* ^2^	9.0%			
Work performance				
DL → WRPT2	0.039	0.023	0.070	0.580
Leader gender → WRPT2	-0.043	-0.052	0.041	0.290
INT → WRPT2	-0.064	-0.077	0.041	0.120
*R* ^2^	2.6%			
Turnover intentions				
DL → TOIT2	0.329	0.368	0.062	0.000
Leader gender → TOIT2	0.020	0.046	0.037	0.596
INT → TOIT2	-0.040	-0.082	0.036	0.260
*R* ^2^	14.3%			

Model, Akaike (AIC) = 77875.134, Bayesian (BIC) = 78943.863,

BOT2 = burnout, TOIT2 = turnover intentions, WRPT2 = work performance, JST2 = job satisfaction, DL = global destructive leadership,

INT = gender as moderator on DL, 0 = male, 1 = female

We found partial support for Hypothesis 4 in that the gender composition of the leader–follower dyad moderates the relation between destructive leadership and follower outcomes (see Table 4). Specifically, the positive relation between destructive leadership and turnover intention was stronger for employees with a gender match (*β* = 0.106, *SE* = 0.037, *p* =.005).

**Table 4:** Regression coefficients from the structural models predicting level at T2, gender match

	Standardized Estimate	Unstandardized Estimate	SE	*p*
Burnout				
DL → BOT2	0.235	0.199	0.074	0.002
GENMATCH → BOT2	-0.016	-0.029	0.035	0.647
INT → BOT2	0.036	0.066	0.039	0.350
*R* ^2^	14.7%			
Job satisfaction				
DL → JST2	-0.168	-0.147	0.081	0.038
GENMATCH → JST2	0.005	0.009	0.035	0.891
INT → JST2	-0.057	-0.108	0.043	0.180
*R* ^2^	8.9%			
Work performance				
DL → WRPT2	0.033	0.019	0.094	0.725
GENMATCH → WRPT2	0.019	0.024	0.039	0.616
INT → WRPT2	-0.049	-0.062	0.048	0.307
*R* ^2^	2.2%			
Turnover intentions				
DL → TOIT2	0.106	0.118	0.071	0.136
GENMATCH → TOIT2	0.000	0.001	0.034	0.992
INT → TOIT2	0.106	0.254	0.037	0.005
*R* ^2^	14.2%			

Model, Akaike (AIC) = 77874.043, Bayesian (BIC) = 788523.772,

BOT2 = burnout, TOIT2 = turnover intentions, WRPT2 = work performance, JST2 = job satisfaction, DL = global destructive leadership, INT = gender match x destructive leadership

Gender: 0 = male, 1 = female

## Discussion

The aim of the present study was to investigate the influence of destructive leadership behavior on followers’ job satisfaction, burnout, work performance, and turnover intention. The aim of this study was also to apply a gender perspective to investigate the role of gender in the destructive leadership process. We were particularly interested in how followers perceive destructive leadership regarding gender and gender match in leader–follower dyads.

In line with previous research, our results confirmed the relations between destructive leadership behavior and follower burnout (Hypothesis 1a), job satisfaction (Hypothesis 1b) and turnover intention (Hypothesis 1d). However, in contrast to previous research [7, 44], we did not find any significant result for destructive leadership behavior and work performance [Hypothesis 1c; 76]. We included both passive and active destructive leadership behaviors in the global factor of destructive leadership. Previous research has argued for passive leadership behaviors to not be seen as always inappropriate, depending on context even positive and empowering, which could explain our results for work performance [77].

Furthermore, our findings did not indicate that a leader’s gender influences follower perceptions of destructive leadership behavior (Hypothesis 2). Additionally, our study showed that leader gender did not moderate the relation between destructive leadership behavior and follower outcomes (Hypothesis 3). Our results contrast with a study on destructive leadership with a German sample of followers [4], where male leaders had a stronger effect on follower health than female ones. Previous studies have shown small or no difference in behaviors between male and female leaders [2, 3]. However, a female leader is perceived different compared to a male one [1]. The perception of gendered behaviors is often connected to cultural context, and according to the Gender Equality Index, Germany was 11th, and Sweden was first in the EU. The Gender Equality Index is a measure that evaluates and compares gender equality across various dimensions, such as economic participation, education, health, and political empowerment. The index typically ranges from 0 (no gender equality) to 100 (complete gender equality) [European Institute for Gender Equality, 78]. The different contexts of Germany and Sweden, where German society is more hierarchical and less gender equal than Swedish society, could partly explain the absence of differences between male and female leaders in this study.

We found partial support for our hypothesis that gender match influences the relationship between destructive leadership and follower outcomes (Hypothesis 4). Specifically, the positive relation between destructive leadership behavior and turnover intention was stronger when the leader and follower were of the same sex than when there was no gender match. Our results suggest that gender similarity amplifies the relation between destructive leadership behavior and follower turnover intention. Previous research has indicated that a lack of perceived leader–supervisor support increases the risk of follower turnover [79]. Empowerment, job satisfaction, and organizational commitment are strong predictors of turnover intention [80]. Sluss and Thompson [81] argued that a follower’s definition of him- or herself at work is closely related to his or her relationship with his or her leader. If supporters are treated disrespectfully, arrogantly, or rudely by their leaders, the followers’ sense of belonging and dedication to the organization is reduced. Being mistreated by the leader is perceived as badly treated by the organization [82]. Demographic similarities can reinforce feelings of betrayal if a leader with whom one identifies acts destructively toward oneself [83] and quitting a job to avoid a threat, such as unsatisfying work conditions, is a well-known coping strategy [84]. One explanation for our results could be that healthy working conditions can buffer the potential negative effect of destructive leadership behavior on follower burnout, job satisfaction, and/or work performance [85]. Organizations that can handle misconduct, such as destructive leaders, within existing structures (e.g., through work environment management and union–employer relations), can hopefully avoid destructive leadership behavior that affects followers’ well-being, work performance, and turnover intention. However, those who can leave their jobs do so, and those who cannot leave can use existing structures within the organization to distance themselves from the destructive leader.

### Implications

From a research perspective, our study contributes to the field of leadership research by investigating destructive leadership behavior and gender through the combination of theory from leadership research with feminist theory. Gendered organization theory contributes to the understanding and knowledge of how organizational structures and contexts, such as organizing processes, organizational culture, interactions between leaders and followers, and individual identity, often preserve and recreate how gender is perceived. Interaction between leaders and followers occurs in the workplace context based on organizational logic [21]. In leadership research, role congruity theory is the dominating theory when investigating discrimination and prejudice connected to gender. Gender role theories can be useful for analyzing how leaders and leadership behavior are perceived by followers. However, to extend the knowledge and understanding of leader–follower interactions, destructive leadership research should incorporate perspectives from feminist research [23]. We consider the construction of gender and leader identity as an ongoing process in relation to others, not just a “role” developed during childhood. Identity is a process occurring within the context of both society and gendered organizational structures, explaining the power and agency asymmetry between male and female leaders. An intersectional feminist research approach could enrich and expand perspectives in the field of feminist leadership studies by recognizing how different social categories intersect to shape unique leadership experiences [16].

From a practical perspective, given the high prevalence of destructive leadership, which was shown to be 36% among workers in Sweden [9], destructive leadership is a serious problem for many organizations. Organizations must work proactively with managers’ work environment to prevent destructive leadership behavior from occurring. This can be achieved through leadership education, but it is equally important for organizations to take responsibility for the work environment of their leaders. Providing appropriate resources and support is essential to enable leaders to effectively manage their day-to-day responsibilities and mitigate stress to prevent destructive leadership behaviors [86]. The relation between managers and employees always takes place in the context of the workplace. Many sectors are sex segregated, which increases possible gender-match interactions between managers and employees, and as this study shows, such gender match may augment the relation between destructive leadership and outcomes. Experiencing destructive leadership behavior from someone we identify with may enhance the negative effect on some outcomes. Our results indicate that gender match between managers and employees can increase the effect of destructive leadership behaviors on turnover intention. However, our results also showed no differences between male and female managers regarding how employees perceive destructive leadership or how destructive leadership affects employee well-being, work performance, and burnout. This indicates a complexity in the leader–follower relationship that cannot be limited to investigate sex differences.

### Strengths and limitations and future research

The combination of gendered organization theory and COR theory adds theoretical complexity and depth that, to the best of our knowledge, is lacking and needs development within destructive leadership research.

A few limitations need to be considered when evaluating the implications of this study. First, the 6-month period between data collection points might be the most important. Little is known about how much time is required between the two time points to capture the effects of the phenomenon of interest [87]. This is also the case for the effects of destructive leadership. A time span of 6 months could be too short or long to adequately capture the potential effect of destructive leadership behaviors on followers’ level of job satisfaction, burnout, work performance, and turnover intention.

Second, this study is vulnerable to common-method bias because we rely on self-reported data. However, in our study, leaders’ destructive behaviors were rated by employees, which could be viewed as an advantage in minimizing self-serving bias compared with leaders’ self-ratings of their behaviors. Hence, employees’ self-reports of their leaders’ destructive leadership behaviors could also be biased by factors affecting their expectations, such as their relation to the leader, demographic characteristics [1, 83], and the amount of information they use to base judgments [88]. The data collection was performed at two separate timepoints. The use of data for the dependent variables in the second and independent variables from the first timepoint decreases the risk of common method bias. We suggest that researchers in future studies use longitudinal study designs with at least three waves over an extended period and capture both the short- and long-term effects of destructive leadership behaviors.

Third, from a gender research perspective, the analysis in this study is limited to using only sex as the gender aspect. The relationship between leaders and followers is reciprocal, and different variables affect this relationship. Leadership is a gendered behavior. For example, leaders exercise power by creating and sustaining cultural values and norms [88]. Think leader, think male is still relevant due to the close connection between masculinities and leader behaviors [89, 90]. Future research utilizing a gender-focused approach should incorporate the concepts of performativity, masculinities, and power dynamics within the context of destructive leadership. This would enhance the understanding of how both leaders’ and followers’ behaviors interact to influence follower outcomes resulting from destructive leadership practices. Additionally, expanding the scope to include the experiences of trans and non-binary leaders and followers would provide valuable insights, offering a more comprehensive understanding of how diverse gender identities intersect with leadership dynamics and the impact of destructive leadership on various groups.

## Conclusion

Like in past research, our study confirms that destructive leadership behavior negatively affects follower outcomes. Our study contributes new knowledge by illuminating how gender affects this process. The construction of gender demonstrates that this is a more complex process than just comparing male and female leaders and that follower gender is also important to consider in this process. In our study, gender match proved to be of relevance. Also, we know from previous research that leaders are treated differently based on gender [10]. To gain a fuller understanding of how various elements of the leader–follower relationship affects follower outcomes, destructive leadership research needs to move beyond the one-sided focus on leaders. By applying gendered organization theory [21, 25], we contribute feminist perspectives to study this phenomenon from new perspectives and formulate further research questions to broaden our scope to include how followers affect destructive leadership behavior, as well as what kind of consequences this has on follower outcomes [23]. Additionally, shifting focus from leader gender to other factors is important for understanding the dimensions of inequality within the destructive leadership phenomenon. We therefore propose to include aspects of organizational context, power, and both sides of the leader–follower dyad in future destructive leadership research.

## Data Availability

The data are available from the authors upon request.

## Declarations

### Declaration of generative artificial intelligence (AI)

Microsoft CoPilot, based on the GPT-4 architecture, was used for enhancing the grammar and wording of the manuscript before submission.
